# Prophylactic Unfractionated Subcutaneous Heparin Does Not Increase Postoperative Hemorrhage in Elderly Patients Undergoing Emergency Neurosurgical Procedures

**DOI:** 10.7759/cureus.24324

**Published:** 2022-04-20

**Authors:** Willians Tambo, Danielle Aronowitz, Ivan Sisa, Erick Diaz, Andrew Y Lee, Joaquin A Cagliani, Fernando J Torres, Rafael Barrera

**Affiliations:** 1 General Surgery, Northwell Health, New York City, USA; 2 Medicine, Universidad San Francisco de Quito, Quito, ECU; 3 Medicine, Universidad Internacional del Ecuador, Quito, ECU; 4 Surgery, Donald and Barbara Zucker School of Medicine at Hofstra/Northwell, New Hyde Park, USA; 5 Anesthesiology, State University of New York Downstate Medical Center, New York, USA; 6 Internal Medicine, Universidad San Francisco de Quito, Quito, ECU

**Keywords:** hemorrhage, pulmonary embolism (pe), subcutaneous unfractionated heparin, emergency neurosurgery, thromboprophylaxis, critically ill elderly patients

## Abstract

Purpose

The purpose of this study is to evaluate the impact in the development of intracerebral hemorrhage in elderly critically ill patients who received prophylactic subcutaneous unfractionated heparin (SCUFH) less than 24 hours after undergoing emergency neurosurgery.

Methods

A retrospective analysis was performed on patients who underwent emergency neurosurgery and were admitted to the surgical intensive care unit (SICU) at a tertiary care center over a 10-year period. Administration of prophylactic SCUFH within 24 hours of neurosurgery was required for inclusion. Demographic and clinical characteristics were recorded. The primary outcome was a rate of postoperative hemorrhagic complications with respect to age.

Results

We identified 223 emergency neurosurgical patients: 100 (45%) patients did not receive prophylactic SCUFH and were excluded. The remaining 123 (55%) patients met all inclusion criteria, of whom 73 (59%) patients were under 65 years old, and 50 (41%) patients were over 65 years old. Patients under 65 years old had significantly lower body mass index (BMI), lower Acute Physiology and Chronic Health Evaluation (APACHE) II, APACHE III, and Simplified Acute Physiology Score (SAPS) scores, and shorter median SICU length of stay compared to patients over 65 years old. No statistically significant difference in the rate of postoperative hemorrhagic or non-hemorrhagic neurological complications was observed between patients in either age group.

Conclusion

Age over 65 years was not associated with a higher risk of postoperative hemorrhage in patients who received SCUFH after emergency neurosurgery. SCUFH can be safely used as a chemoprophylactic agent against venous thromboembolism for elderly patients when used within 24 hours after emergency neurosurgery.

## Introduction

Venous thromboembolism (VTE), including deep vein thrombosis (DVT) and pulmonary embolism (PE), is the third most common cardiovascular condition after myocardial infarction and stroke [1]. Estimates of the global annual incidence of VTE (PE, DVT, or both) range from 45-269 per 100,000 population [1-3]. Rates of VTE formation are higher in men than women, and the risk of VTE increases with age in both groups [2-4]. Although recent global estimates of mortality from VTE are unavailable, VTE is known to be a leading cause of preventable hospital deaths [4, 5]. 

Advanced age is one of several acquired risk factors for VTE [3-7]. Age-specific factors that contribute to the risk of VTE in elderly patients include decreased muscle strength, endothelial dysfunction (reduced vasodilation and reduced intrinsic anticoagulant properties), and progressive decline in venous valve integrity [6]. In addition to advanced age, conventional risks factors for VTE include male sex, diagnosis of malignancy, diagnosis of heart failure, and history of surgery or trauma. In the presence of the risk factors, hospitalized adult patients, and specifically patients admitted to the intensive care unit (ICU), have a high risk of thrombotic events [7]. The risk of VTE is further enhanced among ICU patients due to prolonged immobilization, sepsis, and frequent instrumentation of the central veins for indwelling central venous catheters or other invasive monitoring devices [1, 7].

Subcutaneous unfractionated heparin (SCUFH) is frequently used as primary chemoprophylaxis to prevent VTE in high-risk, hospitalized patients [8], but its use may be contraindicated in patients with increased risk of bleeding complications. The neurosurgical patient is one such example in whom the potential prophylactic benefits of SCUFH may be outweighed by the risk of postoperative intracranial hemorrhage. Two studies have evaluated whether the administration of prophylactic SCUFH is safe for neurosurgical patients [9, 10]. Both studies showed that receipt of prophylactic SCUFH among neurosurgical patients in the early postoperative period was not an independent risk factor for postoperative intracranial bleeding [9, 10].

Given that the incidence of VTE increases with age, we chose to investigate whether age is an independent risk factor for postoperative bleeding complications in a neurosurgical population. To date, there is insufficient data regarding the association between patient age and postoperative bleeding complications in the setting of SCUFH prophylaxis. Thus, the objective of this study was to assess the association of age and the development of postoperative intracranial bleeding complications in patients who underwent emergent neurosurgery procedures and received SCUFH prophylaxis.

## Materials and methods

A retrospective chart review of prospectively acquired data was conducted in all postoperative neurosurgical patients admitted to the surgical intensive care unit (SICU) at a single tertiary center. Inclusion criteria were history of emergency neurosurgery and receipt of SCUFH for DVT prophylaxis within 24 hours of or greater than 24 hours after neurosurgery. Exclusion criteria were lack of prophylactic SCUFH treatment, history of heparin-induced thrombocytopenia (HITT), PE, or DVT. Patients were stratified according to their age as follows: under 65 years old (<65yr) or above 65 years old (>65yr).

All patients in this study were admitted to a neurosurgical attending and managed in the SICU by a dedicated surgical critical care team. All the patients in this study received 5000 units of SCUFH every eight hours from the onset of treatment until discharge. The dosage was selected based on our hospital policy and prior meta-analysis [11-13]. No other dosage was chosen, nor was it changed to another pharmacological agent. In addition, patients routinely received lower extremity intermittent compression boots, and compliance was monitored by nursing staff according to hospital policy. The patients were followed until the date of discharge from the SICU or death.

Sociodemographic data such as age, sex, weight, height, and body mass index (BMI) were collected from the medical record. Illness severity scores, including Acute Physiology and Chronic Health Evaluation (APACHE) II score, APACHE III, Simplified Acute Physiology Score (SAPS), multiple organ dysfunction score (MODS), and length of stay (LOS) in the SICU, were calculated using inpatient data. Outcomes were compared between both age groups (<65yr versus >65yr). The primary outcome was the rate of postoperative intracranial bleeding. The secondary outcome was the rate of DVT, PE, cerebrovascular accident (CVA), cerebrovascular vasospasms, and other neurological complications. All outcomes were evaluated clinically and/or with computed axial tomography (CT) scan. CT scan was ordered only when a careful neurological evaluation yielded a high index of suspicion for intracranial bleeding or an acute change in mental status. As is the policy of the hospital, routine DVT screening with Doppler ultrasounds was not performed, and evaluation of DVT with this study modality was only performed if the patient showed signs and symptoms of DVT.

Descriptive statistics were used to summarize the baseline characteristics of the study participants. Continuous variables were described as mean (standard deviation) or median (interquartile range), and categorical variables, as counts and percentages. Parametric and non-parametric tests were used to evaluate the distribution of categorical and continuous variables by age. A χ2 test was used to assess the relationship between age and the development of postoperative hemorrhagic complications. A two-tailed p-value of less than 0.05 was used to claim statistical significance. All statistical analyses were conducted using R studio version 3.6.2 (R Foundation, Vienna, Austria).

## Results

A total of 223 emergency neurosurgical patients met inclusion criteria. One hundred patients (44.8%) received no anticoagulation, and 123 patients (55.2%) received SCUFH either within 24 hours of or more than 24 hours after neurosurgery. Seventy-three (59.3%) patients were <65yr and 50 (40.7%) patients were >65yr. Sociodemographic data, clinical characteristics, and admitting diagnosis are detailed in Table 1.

**Table 1:** Demographic, clinical characteristics, and admitting diagnosis of neurosurgical patients admitted to the ICU by age SCUFH: subcutaneous unfractionated heparin; BMI: body mass index; APACHE: Acute Physiology and Chronic Health Evaluation; SAPS: Simplified Acute Physiology Score; MODS: Multiple Organ Dysfunction Score; SICU: surgical intensive care unit; LOS: length of stay, IQR: interquartile range; NS: non-significant * Other: non-hemorrhagic, including ischemic

Parameters		<65-year-old patient, n=73	>65-year-old patient, n=50	p-value
Sex	Male, n (%)	27 (37)	19 (38)	1
	Female, n (%)	46 (63)	31 (62)	
Ethnicity	African American, n (%)	6 (8.2)	1 (2.0)	0.37
	Asian, n (%)	8 (10.9)	5 (10.0)	
	Caucasian, n (%)	42 (57.5)	37 (74)	
	Hispanic, n (%)	14 (19.1)	6 (12)	
	Other, n (%)	3 (4.1)	1 (2)	
BMI, Kg/m2	Normal: 18.5-24.9, n (%)	39 (54.2)	30 (61.2)	0.01
	Overweight: 25-29.9, n (%)	17 (23.6)	17 (34.7)	
	Obese: ≥ 30.0 (%)	16 (22.2)	2 (4.1)	
SCUFH <24h	APACHE II, median (IQR)	8 (5-11)	11.5 (9-16.5)	<0.001
	APACHE III, median (IQR)	25.5 (15.5-40.5)	45.5 (36-60)	<0.001
	SAPS, median (IQR)	21 (15.25-26)	33 (28.25-42)	<0.001
	SICU LOS, days median (IQR)	7 (3-12.75)	2 (2-4.75)	<0.001
SCUFH >24h	APACHE II, median (IQR)	6 (4.5-7.5)	10.5 (9-12.25)	<0.01
	APACHE III, median (IQR)	22 (14.5-30)	36 (34-41.25)	<0.001
	SAPS, median (IQR)	20 (16-22.5)	30.5 (29.75-34)	<0.001
	SICU LOS, days median (IQR)	5 (3-11.5)	12 (3.75-13)	0.3106
Admitting diagnosis	Subarachnoid hemorrhage, n (%)	39 (53.4)	16 (32)	0.001
	Intracerebral hemorrhage, n (%)	15 (20.5)	9 (18)	
	Subdural hemorrhage, n (%)	10 (13.6)	7 (14)	
	Other, n (%) *	9 (12.5)	18 (36)	

We found no statistically significant difference between sex and ethnicity stratified by age. Patients <65yr were less likely to have normal BMI than patients >65yr (p<0.05); likewise, a greater proportion of patients <65yr were obese compared to patients >65yr. The following admitting diagnoses were all more common in patients <65yr than in patients >65yr: subarachnoid hemorrhage (53.4% vs. 32%), intracerebral hemorrhage (20.5% vs. 18%), and subdural hemorrhage (13.6% vs. 14%; p=0.001). Illness severity scores on admission (APACHE II, APACHE III, and SAPS scores) were higher in the >65yr group (p<0.01). Further, median SICU LOS was longer in patients <65yr compared to patients >65yr (seven days vs. two days, p<0.001).

A total of 34 patients experienced neurologic complications: 29 patients were <65yr and five patients >65yr. We found no statistically significant difference in the rate of these complications by age. The most frequent non-hemorrhagic complications were cerebrovascular accidents and vasospasm (Table 2). 

**Table 2:** Frequency of postoperative complications in post-neurosurgical patients by age SAH: subarachnoid hemorrhage; CVA: cerebrovascular accident; CVV: cerebrovascular vasospasm *Altered mental status, disseminated intravascular coagulation, paraplegia, thrombophlebitis, ventriculitis

Parameter		<65 years old n=29	>65 years old n=5	p-value
Hemorrhagic	SAH re-bleeding, n (%)	1 (3.4)	0	1
Non-hemorrhagic	CVA, n (%)	10 (34.5)	1 (20)	0.08
	CVV, n (%)	13 (44.8)	3 (60)	0.06
	Other, n (%)*	5 (17.3)	1 (20)	1

## Discussion

The present study aimed to evaluate the impact of age (<65yr versus >65yr) on the incidence of postoperative intracranial hemorrhagic complications in neurosurgical patients who received prophylactic SCUFH either within 24 hours of or greater than 24 hours after neurosurgery. We did not find an association between postoperative intracranial bleeding and age. Our findings suggest that age is not an independent risk factor for developing hemorrhagic complications in the setting of SCUFH prophylaxis after emergency neurosurgery. These findings further support previous studies that have reported the use of prophylactic SCUFH within 24 hours of emergency neurosurgery without increased risk of postoperative bleeding [9, 10].

While studying the characteristics of our cohort, we observed that there was a greater proportion of obese patients in the <65yr group. Rates of cerebrovascular accident, cerebrovascular vasospasm, and other postoperative neurological complications (paraplegia, thrombophlebitis, ventriculitis) were higher in the <65yr group, and patients in this group had longer median SICU LOS. These findings are consistent with previous reports that have linked obesity with stroke risk and adverse ICU outcomes [14, 15]. Illness severity scores such as APACHE II, APACHE III, and SAPS were higher in the >65yr group.

The main limitation of the present study is its retrospective nature and small sample size. There was only one case of postoperative intracranial bleeding in our cohort, which yields insufficient power to statistically test for causality between SCUFH administration and postoperative complications. Despite this, our findings are in accordance with the present evidence that has reassured the safety of SCUFH administration in emergency neurosurgical patients [9, 10].

## Conclusions

Based on the present analysis, the administration of SCUFH as DVT prophylaxis is not associated with higher rates of postoperative hemorrhagic complications in elderly patients. This contributes to the knowledge that SCUFH can be used as DVT prophylaxis even in elderly emergency neurosurgical patients. Future longitudinal and multicenter studies are needed to further inform clinical practice.

## References

[1] Miri M, Goharani R, Sistanizad M (2017). Deep vein thrombosis among intensive care unit patients; an epidemiologic study. Emerg (Tehran).

[2] Shaikhouni A, Baum J, Lonser RR (2018). Deep vein thrombosis prophylaxis in the neurosurgical patient. Neurosurg Clin N Am.

[3] Wendelboe AM, Raskob GE (2016). Global burden of thrombosis: epidemiologic aspects. Circ Res.

[4] Grosse SD, Nelson RE, Nyarko KA, Richardson LC, Raskob GE (2016). The economic burden of incident venous thromboembolism in the United States: a review of estimated attributable healthcare costs. Thromb Res.

[5] Heit JA (2015). Epidemiology of venous thromboembolism. Nat Rev Cardiol.

[6] Engbers MJ, van Hylckama Vlieg A, Rosendaal FR (2010). Venous thrombosis in the elderly: incidence, risk factors and risk groups. J Thromb Haemost.

[7] Ejaz A, Ahmed MM, Tasleem A (2018). Thromboprophylaxis in intensive care unit patients: a literature review. Cureus.

[8] Yayan J, Bals R (2016). Relative risk of deep vein thrombosis in very elderly patients compared with elderly patients. Clin Appl Thromb Hemost.

[9] Hacker RI, Ritter G, Nelson C (2012). Subcutaneous heparin does not increase postoperative complications in neurosurgical patients: an institutional experience. J Crit Care.

[10] Cagliani J, Ritter G, Nelson C (2017). Bleeding risk and thromboprophylaxis in neurosurgical patients after emergency procedures. J Intensive & Crit Care.

[11] Alhazzani W, Lim W, Jaeschke RZ, Murad MH, Cade J, Cook DJ (2013). Heparin thromboprophylaxis in medical-surgical critically ill patients: a systematic review and meta-analysis of randomized trials. Crit Care Med.

[12] Park J, Lee JM, Lee JS, Cho YJ (2016). Pharmacological and mechanical thromboprophylaxis in critically ill patients: a network meta-analysis of 12 trials. J Korean Med Sci.

[13] Cook D, Meade M, Guyatt G (2011). Dalteparin versus unfractionated heparin in critically ill patients. N Engl J Med.

[14] Kernan WN, Inzucchi SE, Sawan C, Macko RF, Furie KL (2013). Obesity: a stubbornly obvious target for stroke prevention. Stroke.

[15] Wardell S, Wall A, Bryce R, Gjevre JA, Laframboise K, Reid JK (2015). The association between obesity and outcomes in critically ill patients. Can Respir J.

